# Optimizing the safety-efficiency trade-off on nationwide air traffic network flow using cooperative co-evolutionary paradigm

**DOI:** 10.1038/s41598-025-08375-7

**Published:** 2025-07-01

**Authors:** Mingming Xiao, Chen Hong, Kaiquan Cai

**Affiliations:** 1 College of Smart City, Beijing Union University, Beijing, 100101 China; 2 Beijing Key Laboratory of Information Service Engineering, Beijing Union University, Beijing, 100101 China; 3 College of Robotics, Beijing Union University, Beijing, 100101 China; 4 School of Electronics and Information Engineering, Beihang University, Beijing, 100191 China

**Keywords:** Air traffic network flow optimization, Large-scale, Multi-objective optimization, Cooperative co-evolution, Mathematics and computing, Computational science

## Abstract

Safety and efficiency are two classical conflicting objectives in the air traffic system: an increase in efficiency may come at the cost of increasing density of aircraft in the space, which increases collision risk and controllers’ workload. Nationwide air traffic network flow optimization (ATNFO) is an effective way to pursue trade-offs between safety and efficiency by optimizing flight departure time-slots and routes within a given time period and under the latest airspace resources. Solving a national ATNFO problem is usually bedeviled by “the curse of dimensionality” as it consists of a huge number of variables. This paper presents a specific “divide-and-conquer” based approach, namely H-COEA, to solve it. Firstly, an effective chromosome representative scheme, which can be naturally divided into 3 sub-components, i.e., the departure time-slots, the heuristic for selecting flight route, and the timetabling indicating the order and fairness for flight to select route, is employed. And then, the corresponding 3 sub-populations are co-evolved based on a Cooperative Co-evolution (CC) paradigm. Four different-scale ATNFO problems are solved with H-COEA and the state-of-the-art multi-objective evolutionary algorithms. Results show that H-COEA obtains better trade-offs between safety and efficiency, making CC paradigm appropriating for solving large-scale ATNFO problem.

## Introduction

Due to the rapid development of global air transportation, airspace traffic congestion and flight delays have become more and more severe and placed increasing strain on the Air Traffic Flow Management (ATFM) system, particularly in China, where the number of flight operations has doubled over the last decades and the ratio of delayed flights has reached 20.09% in 2024. Hence, air traffic flow optimization, which aims to reduce the occurrence of congestion and delays by optimizing the allocation of airspace resources (i.e., airport, sector, and time-slot) to safely accommodate the traffic demand before operation, has become a hot research topic in ATFM domain^1^.

In recent years, novel concepts and technologies have been proposed to support the future ATM system^2^. Trajectory-based operations (TBO) is a key enabler of several operational improvements planned for NextGen^3^ and SESAR^4^, and become the future trend in the global world^5,6^. TBO seeks to improve the predictability of the ATM system as a whole in order to increase capacity and efficiency while maintaining safety. The TBO concept consists of the use of 4-dimensional trajectory (4DT), i.e., flight path in three-dimensional space and time, as the basis for efficient planning, coordinating, and executing flight operations. Generating optimal 4DT data for all scheduled flights from a global view during the pre-tactical air traffic flow management phase is therefore necessary and essential for TBO.

Hence, the nationwide air traffic network flow optimization (ATNFO) problem, which aims to keep sure air traffic safety and efficiency during the whole operational phase by constructing optimal flight plans for all scheduled flights over a period of time horizon (usually 1–24 h) based on latest capacity information, has drawn significant attention recently^7–9^. Here a flight plan is a collection of information including flight route, departure time-slot, arrival time-slot and time-slots of entering and exiting sectors, i.e., 4DT data of a flight.

To solve a nationwide ATNFO problem is challenging. Firstly, ATNFO essentially consists of two coupled sub-problems, i.e., the flight scheduling problem for deciding optimal departure time-slot and the flight rerouting problem for deciding optimal flight route over airspace. Secondly, a large number of flights flying over the national airspace are needed to consider, especially in peak hours.

In previous studies, most work considered the solely flight delays and formulated a single-objective mathematical programming model for ATNFO problem. For example, Bertsimas and Odoni^10^ proposed a basic 0–1 integer programming (IP) model for ATNFO problem to optimize departure and arrival time-slots for each aircraft with the objective of minimizing total flight delay costs, while satisfying airports and sectors capacity constraints. The 0–1 IP model was solved by linear programming relaxation. Later, continued improved IP models have been proposed to cover all the phases of each flight (takeoff, en-route cruising and landing) and allow flight rerouting in the ATNFO problem, such as BLO model (Bertsimas, Lulli and Odoni)^11^ and mixed IP (MIP) model (Agustin et al.)^12,13^. There are also IP models for ATNFO problem based on aggregated traffic flow^14^ and solved with dual decomposition method^15^, which reduce the problem dimension but lose individual flight’s information.

While the above research has extensively modeled delays and efficiency in air traffic networks, the integration of safety indicators, such as collision risk probability^16^ and sector workload variability^8,17,18^, remains underexplored. Studies like Zhang et al.^19^ have used Bayesian networks to quantify safety risks in airport clusters, but few have operationalized these metrics into real-time optimization frameworks. This study addresses this gap by developing a hybrid model that incorporates sector workload, as defined in ICAO’s Safety Management System (SMS)^20,21^, into a multi-objective optimization framework for flight routing. By balancing delay minimization with safety-risk penalties, our approach aims to operationalize the safety-efficiency frontier concept for national air traffic systems.

Therefore, an ATNFO problem, by its very nature, is a pursuit of trade-offs between air traffic safety and efficiency, i.e., simultaneously minimization of air traffic control (ATC) workload and flight delays, within limited resources. In ATFM system, all airports and en-route sectors are capacitated entities due to capacity limitations imposed because of ATC workload^22^. The more workload allowed, the more flights can simultaneously operate in the airspace, resulting in less flight delay. Likewise, the more flight delays allowed, the less airspace congestion emerging and less ATC workload needed. The comprehensive quantification of ATC workload is expressed in nonlinear function^8,17^, which can hardly be solved by mathematical programming methods.

An attractive work was presented in Ref.^17^ by Daniel et al. In that work, they proposed a generalized model to simultaneously minimize the ATC workload and flight delays, and obtained optimal departure time-slot and route within a predefined set using multi-objective genetic algorithm (MOGA)^23^, which sparks a new line of research ATNFO problem from the perspective of stochastic optimization. Evolutionary algorithms (EAs) usually work well on stochastic optimization problems^24,25^. For an ATNFO problem, there is always a high-dimensional search space since thousands of flights to be scheduled a day and at least two decision variables (i.e., departure time-slot and flight route) needed to be optimized for a flight. Hence, the application of traditional EAs to ATNFO problem will suffer from the “curse of dimensionality”. Recently, a Cooperative Co-evolution (CC) paradigm^26,27^, which adopts the idea of “divided and conquered” to divide a problem into two or more low-dimensional sub-problems that became easier to deal with, has been successfully embedded to scale EAs up to large-scale and complex problems^28–31^, including solving ATNFO problem^30,31^.

Due to the large-scale and nonlinear characteristics of ATNFO problem, evolutionary algorithms using CC paradigm are more appropriate for seeking the trade-offs between air traffic safety and efficiency, when compared to mathematical programming method. However, for existing evolutionary algorithms for solving ATNFO problem^17,30,31^, the assumption that only a fixed set of alternative routes are available for flight rerouting may become a serious limitation for selecting an optimal route for each flight and lead to lower utilization of airspace capacity. And some fairness should be considered in the flight route allocation^32^.

Motivated by the above analysis, we proposed a hybridized indirect and direct encoding multi-objective cooperative co-evolutionary algorithm (H-COEA) for solving the ATNFO problem. The contributions are two-fold. Firstly, we employed a specific designed heuristic for selecting flight route, inspired by Ref.^8^. It will substitute selecting flight route within a predefined set (the main way of flight rerouting in previous work) in our paper to improve the utility of airspace capacity and smooth the traffic flow over airspace. With the heuristic, an effective chromosome representative scheme, which can be naturally divided into three subcomponents, i.e., the departure time-slots, the heuristic for selecting flight route, and the timetabling indicating the order and fairness for flight to select route, is proposed. It is a hybridized indirect and direct encoding for ATNFO problem: direct representation of departure time-slot and indirect representation of flight route. Then, each sub-population is designed and evolved in the form of interacting with others within a CC paradigm^36^. All sub-populations cooperatively produce complete solutions to the ATNFO problem.

The rest of this paper is organized as follows: Section "Problem formulation" introduces the mathematical model of ATNFO problem. Section "Decision variables" presents the H-COEA for solving ATNFO problem, including the details of heuristic for flight route selecting, sub-population representation and cooperative mechanism. Experiment study is presented in Section "Constraints" to evaluate the effectiveness of our H-COEA algorithm. Finally, Section "Objectives" concludes this paper.

## Problem formulation

The essence of ATNFO problem is to optimize departure and arrival time-slots, as well as flight routes for each flight, while obeying capacity constraints on various airspace entities (sectors and airports) and time constraints on each flight. Its two objectives are minimizing ATC workload and total flight delays, which conflict and should be minimized simultaneously.

In this section, we will describe the mathematical formulation of our ATNFO problem. This heavily upon the bi-objective formulation mentioned in Ref.^8^. Following previous work, our formulation is defined on the basis of three assumptions and principles:

1. The airspace is defined as an air traffic network (ATN) (see Fig. 1), denoted as $G(N,E)$, where $N$ is the set of nodes and $E$ is the set of edges. As we know, airspace is divided into a set of sectors. There are two kinds of nodes, i.e., sectors and airports. Suppose the number of airports and sectors in ATN are $n$ and $m$ respectively, then $N = \{ N_{1} ,...,N_{n} ,N_{n + 1} ,...,N_{n + m} \}$. The edges are interconnecting pairs of nodes. An edge exists between the two sectors in ATN only when these two sectors are physically adjacent in airspace and there are air routes from one sector to another.

**Fig. 1:**
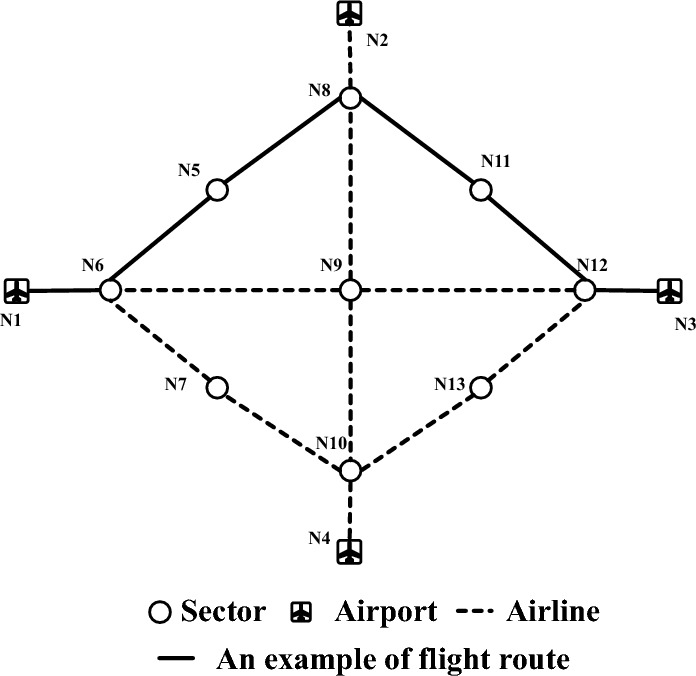
Example of ATN and flight route.
2. When a flight departs, it directly enters into an en-route sector. After crosses a set of continuous sectors, it will arrive at its destination airport. A flight route demonstration can be seen in Fig. 1. This assumption focuses the analysis on en-route dynamics and is consistent with prior studies on air traffic network flow optimization^8^.
3. Each flight flies at a constant speed $v$ (kilometers per hour). Hence, when determining the departure time-slot and route of a flight, we can calculate some other information, such as, arrival time-slot and time-slots of entering and exiting sectors.

Based on above assumptions, various parts of our ATNFO model, i.e., decision variables, constraint sets, and objective functions, are introduced as following.

## Decision variables

Flight plans for all scheduled flights over a period of time horizon (usually 1–24 h) based on latest capacity information are optimized in our ATNFO problem to keep sure air traffic safety and efficiency. Here a flight plan is a collection of information including flight route, departure time-slot, arrival time-slot and time-slots of entering and exiting sectors. Based on assumption 3, two sets of decision variables are used in our ATNFO problem formulation: flight departure time-slots $\{ T_{d}^{1} ,T_{d}^{2} ,...,T_{d}^{f} ,...,T_{d}^{n\_F} \}$ and flight routes $\{ R^{1} ,R^{2} ,...,R^{f} ,...,R^{n\_F} \}$.

Where $F = \{ F_{1} ,F_{2} ,...,F_{f} ,...,F_{n\_F} \}$ are the flight set, i.e., traffic demand, during the given time period *T*, which is divided into discrete, equal-sized and contiguous time-slots and denoted as $\{ 1,2,...,T\}$ in our ATNFO problem. $n\_F$ is the number of flights. $T_{d}^{f}$ is the departure time-slot of flight $f,f \in F$, and is a integer value. $R^{f}$ is the route of flight $f,f \in F$, and $R^{f} = \{ N_{1}^{f} ,N_{2}^{f} ,...,N_{k}^{f} ,...\} ,N_{k}^{f} \in N$, which represents the sequence of continuous sector nodes flight *f* crossed along its route. For each flight, the departure and arrival airports are determined by traffic demand.

## Constraints

Two types of constraints are considered here, one is time constraint, and the other one is capacity constraint. The corresponding three constraints are listed below:

1 $$T_{d}^{f} \in [\underline{T}_{d}^{f} ,\overline{T}_{d}^{f} ],f \in F$$

2 $$\frac{{L(R^{f} )}}{v} \in [t_{\min }^{f} ,t_{\max }^{f} ],f \in F$$

3 $$\begin{gathered} \sum\limits_{f \in F} {g_{i}^{f} (t) \le C_{i} (t)} ,i = 1,...,n + m,t \in T, \hfill \\ g_{i}^{f} (t) = \left\{ {\begin{array}{*{20}c} {1,} & {ifflight f exsitsin nodeN_{i} at time t} \\ {2,} & {otherwise} \\ \end{array} } \right. \hfill \\ \end{gathered}$$

Equation (1) and (2) are time constraints. Equation (1) is departure time-slot constraint for each flight since a flight shouldn’t be delayed or advanced for a long time according to practical traffic operation requirements. $\underline {T}_{d}^{f}$ and $\overline{T}_{d}^{f}$ denote the earliest and latest available departure time-slots of flight *f* respectively. Equation (2) is flight time range for all flights. $t_{\min }^{f}$ is the time spending along the shortest route of flight *f* at constant speed $v$. $t_{\max }^{f}$ is the allowed maximum flight time of flight *f*, it mainly determined by the fuel that aircraft taken. For a domestic flight, $t_{\max }^{f}$ is usually one and a half hour more than $t_{\min }^{f}$.

During the given traffic operation time period, each flight takes off at its departure time-slot and follow its route. The distributions of traffic in airspace are variable over time. For each airspace entity (i.e., sector or airport) in each time-slot, capacity constraint should be obeyed. Equation (3) expresses capacity constraints of all airports and sectors, and the capacity $C_{i} (t)$ of node $N_{i} ,i = 1,2,...,n + m$ is a function related with time *t*. Here, *n* and *m* represent the number of airports and sectors in an ATN respectively. Usually, capacity is represented by number of aircraft that can be safely handled in a given airspace.

## Objectives

The aim of our ATNFO is to pursue a trade-off between air traffic safety and efficiency, i.e., simultaneously minimization of ATC workload and flight delays, within limited resources. That is to say, ATNFO problem has the following two conflict objectives:

Objective 1: minimization of ATC workload (ATCW). During the aircraft operation, air traffic controllers are responsible for keeping the aircraft flying safely and efficiently along their trajectories. And the resulting ATC workload in each node $W_{i} (t),i = 1,2,...,n + m$, which composed of monitoring workload ($W_{i}^{mo}$) and coordination workload ($W_{i}^{co}$)^17^, is closely related to air traffic safety. Because of that, congestion will emerge in overload sectors and airports, and spread over the whole ATN. Here, $W_{i}^{mo}$ is the number of flight that flying in sector *i* during time slot *t*, $W_{i}^{co}$ is the number of flight that entering/exiting sector *i* during time slot *t*. Hence, minimizing the ATCW is essentially to smooth the workload over ATN and minimize the total workload. The objective of minimizing ATCW is defined as following:

4 $$\min ATCW:f_{1} = \sum\limits_{i = 1}^{i = n + m} {((\sum\limits_{t \in T} {W_{i} (t)} )^{1 - \varphi } \times (\mathop {\max }\limits_{t \in T} W_{i} (t))^{\varphi } )}$$

where $\varphi$ is an index between 0 and 1. It assigns weight factors to the total workload and the maximum workload of each sector. $W_{i} (t) = k^{mo} \cdot W_{i}^{mo} (t) + k^{co} \cdot W_{i}^{co} (t)$, $k^{mo}$ and $k^{co}$ are coefficients of monitoring workload and coordination workload, respectively. Since the coordination workload between two sectors are more complicated than monitoring workload in a sector, we have $k^{mo} = 1$ and $k^{co} = 2$. For airports, we just consider workload as the number of departure and arrival flights.

Objective 2: minimization of total flight delays (TFD). The pursuit of minimization of airspace congestion inevitably leads to an increasing of flight delays. The objective of minimizing TFD^17^ can be calculated as following:

5 $$\min TFD:f_{2} = \sum\limits_{f \in F} {\left| {\left| {T_{d}^{f} - T_{do}^{f} } \right| + w \cdot ((T_{a}^{f} - T_{d}^{f} ) - (T_{ao}^{f} - T_{do}^{f} ))} \right|}$$

where $T_{do}^{f} ,T_{ao}^{f}$ are the original scheduled departure and arrival time-slots of flight *f* ($f \in F$) respectively. $T_{d}^{f} ,T_{a}^{f}$ are optimized departure and arrival time-slots of flight *f* ($f \in F$)respectively, and $T_{a}^{f} = T_{d}^{f} + \frac{{L(R^{f} )}}{v}$ ($L(R^{f} )$ is the length of flight route $R^{f}$). $\left| {T_{d}^{f} - T_{d0}^{f} } \right|$ is the ground delay induced by ground-holding. $w \cdot ((T_{a}^{f} - T_{d}^{f} ) - (T_{ao}^{f} - T_{do}^{f} )$ is the airborne delay induced by extra flight distance caused by rerouting, and the index $w(w > 1)$ denotes that airborne delay is more costly than ground delay.

## Cooperative coevolution for ATNFO problem

Recalling that the major attempt of this work is to address the nationwide ATNFO problem, which is characterized by high dimensionality since a large number of flights’ trajectories (composed of departure time-slots and flight routes) are needed to optimize. For example, in the context of the national airspace of China, thousands of flights fly through the airspace in a day and are needed to be considered. Moreover, ATNFO is essentially a non-linear multi-objective combinatorial problem and has been proved to be a NP hard problem^10,17^. Hence, CC strategy^26^, which adopts the idea of “divided and conquered” to divide a problem into two or more low dimensional sub-problems and evolves them cooperatively, is quite suitable for solving ATNFO problem. The integration of solutions from all sub-problems forms a complete solution.

**Fig. 2:**
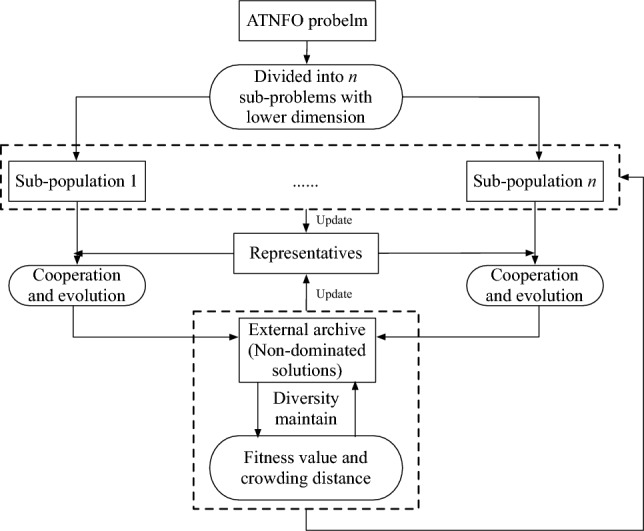
The general framework of H-COEA.

To explore optimized trade-offs between air traffic safety and efficiency, an algorithm using cooperative co-evolution paradigm, i.e., the H-COEA, is proposed in our work. The general framework of H-COEA is shown in Fig. 2. Firstly, the ATNFO problem is divided into several sub-problems to reduce search space, and then the corresponding sub-populations are designed and evolved in the form of interacting with other sub-populations (representatives) within CC paradigm. The challenges are how to identify and represent sub-populations (i.e., sub-problems) for ATNFO problem, and how to co-evolve them.

The following subsections give details of our proposed H-COEA, including population design, collaboration mechanism, and overview of H-COEA for solving the ATNFO problem.

### Population design of ATNFO problem

An ATNFO problem, due to its characteristic of two coupled problems, can be naturally decomposed into two sub-problems, i.e., the flight scheduling problem and the flight rerouting problem.

In order to maximize the utility of airspace capacity and achieve global optimal routes for all scheduled flights, a heuristic is designed for selecting optimal flight routes based on current air traffic operating picture. As we know that, there are usually multiple candidate routes from one airport to another in an ATN. For instance, we can find 9 candidate routes exist between airport *N*_1_ and airport* N*_3_ in Fig. 1. In practical, the number of candidate routes for each pair of airports is much larger. Since all flights share the same limited airspace resource and time resource, any flight route should be selected based on current traffic situation awareness information (i.e., workload) in airspace. And moreover, from the perspective of both safety and efficiency, a good flight route should have less distance and workload, and allow the flight to depart on time. Hence, we defined the optimal route $R_{opt}^{f}$ as following^8^:

6 $$R_{opt}^{f} = \mathop {\min }\limits_{{k = 1,...,n_{r}^{f} }} \left\{ {H_{f} \cdot \sum\limits_{{i \in S_{{R_{k}^{f} }} }} {\frac{{S_{i} }}{{S_{\max } }}} + \left( {1 - H_{f} } \right) \cdot \sum\limits_{{i \in S_{{R_{k}^{f} }} }} {\frac{{W_{i} }}{{W_{\max } }}} } \right\}$$

where, $S_{{R_{k}^{f} }}$ is the set of crossed sectors of route $R_{k}^{f}$, $S_{i}$ is the average crossing distance of sector *i*, $W_{i}$ is the current workload of sector *i* and related to the number of aircraft that crossed sector *i*. $S_{\max } = \mathop {\max }\limits_{i = 1,...,m} \{ S_{i} \}$ and $W_{\max } = \mathop {\max }\limits_{i = 1,...,m} \{ W_{i} \}$ are applied to normalize the length and workload of flight route, and *m* is the number of sectors in ATN. The heuristic weight, $H_{f} \in [0,1][0,1]$, will be evolved to find a better trade-off between safety and efficiency for flight $f$. We can notice that, with extreme values of $H_{f}$, i.e., $H_{f} = 0$ and $H_{f} = 1$, the selected routes correspond to the minimum workload route and minimum distance route respectively.

Based on above heuristic, a timetabling has been applied and evolved to decide the order of a flight to select route, which overcomes the bias that the order of flights for selecting route is fixed and gives fair utilization of airspace resources to each flight. Solving flight rerouting problem can be further decomposed into two sub-problems. One is deciding heuristic for selecting optimal flight route. The other one is deciding order for a flight to select route.

**Fig. 3:**
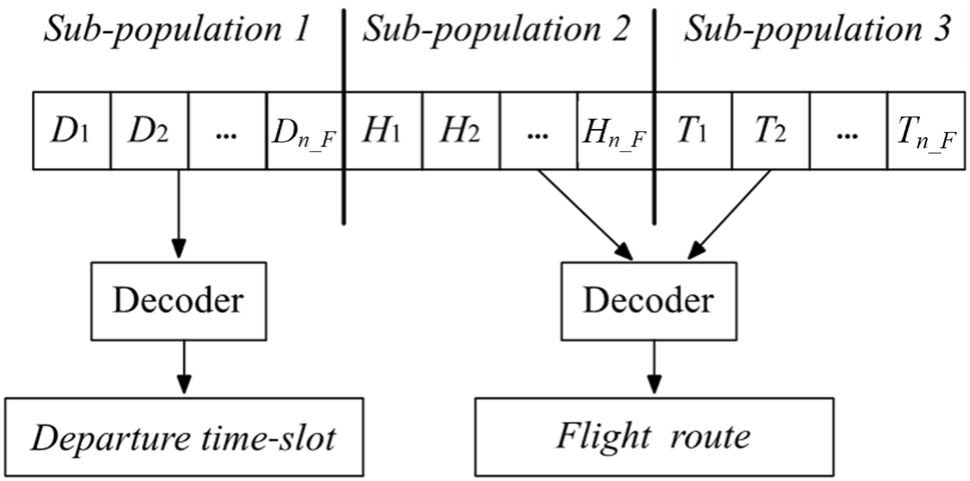
Chromosome design for each sub-population of ATNFO problem.

Hence, an ATNFO problem can be naturally divided into three sub-problems: deciding optimal departure time-slots, optimal heuristics and orders of flight route selecting for all scheduled flights. Each sub-problem is assigned a sub-population. And a Hybridized chromosome representation inspired by Ref.^8^ that uses indirect encoding of flight route and direct encoding of departure time-slot is introduced here. The corresponding chromosome representations of individuals in three sub-populations are designed as Fig. 3.

Each chromosome represents a possible solution to its sub-problem. There are $n\_F$ genes in each chromosome, $n\_F$ is the number of scheduled flights. The gene values are presented as following:

$D_{f}$ is the departure time-slot of flight *f* ($f = 1,2,...,n\_F$). It is integer coded and varies in a predefined time interval $[\underline{T}_{d}^{f} ,\overline{T}_{d}^{f} ]$ (Eq. (4)).

$H_{f}$ is the heuristic weight (introduced in Section "Population design of ATNFO problem") of flight *f* ($f = 1,2,...,n\_F$). It is a continuous value between 0 and 1, and decides the way of selecting flight route, i.e., the trade-off between safety and efficiency for flight.

$\{ T_{1} ,T_{2} ,...,T_{n\_F} \}$ is a timetabling which is responsible for the order of withdrawing a flight to be scheduled using the corresponding heuristic. For example, $T_{f}$ means the route of flight *f* determined after preceding $T_{f} - 1$ flight. This timetabling can overcome the bias that the order of flights is fixed; that is, the first flight with index 1 (i.e., flight *f*, $f = 1$) will always be scheduled first and have priority to occupy available airspace resources.

The decoder in Fig. 3 refers to the mechanism that maps the encoded representations to their corresponding phenotypic values. For sub-population 1, it is integer coded, the genotype directly serves as the phenotype, the decoder translates these integer numbers (genotype) into discrete time-slots (phenotype), i.e., the departure time-slots of all flights. Sub-populations 2 and 3 share the same decoder in this architecture. The decoder translates each heuristic weight (real coded) into a flight route according to Eq. (6) (the definition of the optimal route based on heuristic weight), the timetabling in sub-population 3 decide the translation order.

**Algorithm 1:**
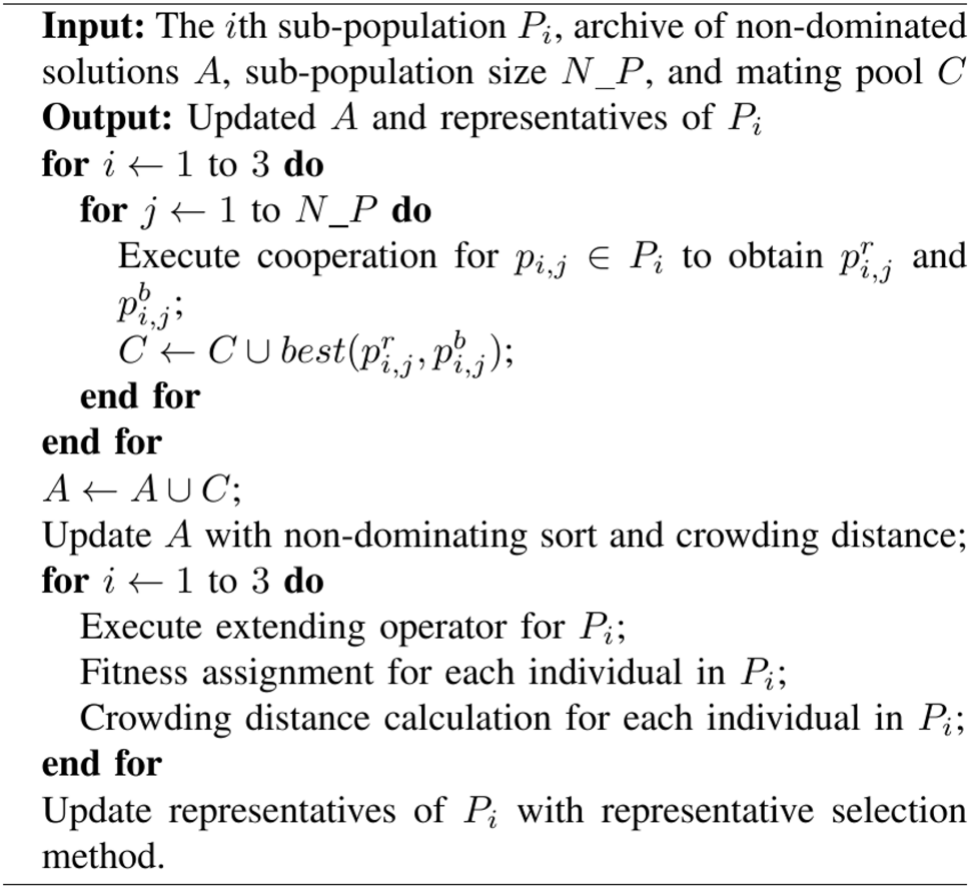
Cooperative process.

### Cooperative mechanism

Having identified a natural decomposition of a problem, CC technique assigns sub-population to each sub-problem, and evolves each sub-population independently and simultaneously with evolutionary algorithms. The critical difference in moving standard evolutionary algorithms into CC framework is that, when evaluating an individual (mainly refers to its extent of cooperation and fitness) from one sub-population, it is necessary to cooperate the individual with representatives selected from the other sub-populations to form a complete candidate solution. An appropriate cooperative mechanism is crucial for exploiting the fine-grained search capability and maintaining good diversity desirable in many applications^33,34^. The pseudocode of cooperative mechanism in our proposed H-COEA algorithm is shown in Algorithm 1. Some details are given as bellowing:

1. Representative selection. In an air traffic flow optimization, any flight’s departure time-slot and route have an effect on some other flights’ plan due to limited time and space resources, which they are sharing in an ATN. And for each flight, there is an interaction effect between its departure time-slot and route. Hence, the decomposed three sub-problems of ATNFO problem (Section "Population design of ATNFO problem") are strongly coupled. Problem landscapes with strong inter-activity between components seem to require less greedy methods for selection of collaborators^35^. To avoid sub-populations being trapped into local optimal, for each sub-population, two representatives are chosen for cooperative^36^. One is randomly selected from the external archive, named random representative and denoted as “R” in Fig. 4. The other one is the best individual in the external archive, named best representative and denoted as “B” in Fig. 4. Here, we consider the one that has the largest crowding distance (except boundary solution) in the external archive as the best individual. A demonstration of selecting representatives can be seen in Fig. 4, in which P_1_, P_2_, and P_3_ denote sub-population 1, 2, and 3 (mentioned in Fig. 3) respectively. An external archive, denoted as *A*, is used to store current obtained non-dominated solutions. At the beginning of evolution, the external archive is empty, and both random representative and best representative are randomly selected from sub-populations.

**Fig. 4:**
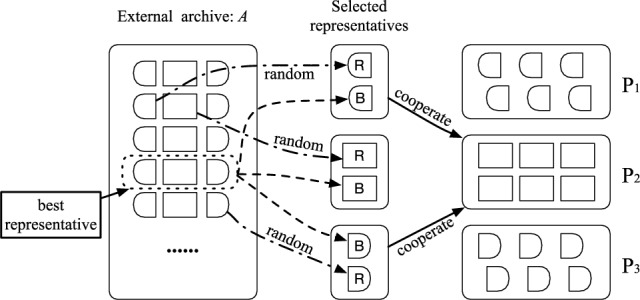
Demonstration of representative selection.
2. Cooperation. Two types of cooperation are implemented to evaluate individuals in each sub-population. One is combining the evaluated individual with random representatives from the other two sub-populations. While the other one is combining the evaluated individual with best representatives from the other two sub-populations. The corresponding obtained complete solutions are denoted as $p_{i,j}^{r}$ and $p_{i,j}^{b}$. The better one will be put in the “mating pool” (*C*) to update the external archive and used to evaluate fitness value. The better one is determined by Eq. (7), where $random(p_{i,j}^{r} ,p_{i,j}^{b} )$ means picking one from $\{ p_{i,j}^{r} ,p_{i,j}^{b} \}$ at random.
  7 $$best(p_{i,j}^{r} ,p_{i,j}^{b} ) = \left\{ {\begin{array}{*{20}c} {p_{i,j}^{r} ,ifp_{i,j}^{r} dominantsp_{i,j}^{b} } \\ {p_{i,j}^{b} ,ifp_{i,j}^{r} dominantsp_{i,j}^{b} } \\ {random(p_{i,j}^{r} ,p_{i,j}^{b} ),otherwise} \\ \end{array} } \right.$$
3. Fitness assignment. An individual’s fitness depends on how many members in the external archive and its sub-population dominate it, as well as how many individuals in its sub-population dominated by it^36^. The steps of calculating fitness value are: firstly, for an individual, cooperate it with representatives to form a complete solution, and determine each flight route in the order of timetabling based on heuristic; Then, calculate its two objectives of ATNFO problem, i.e., the expected ATC workload and total flight delays, according to all flights’ departure time-slots and routes; At last, calculate the fitness value as Eq. (8). Where $N_{i}^{ExArchive}$ and $N_{ia}^{SubPop}$ are the numbers of individuals that dominate individual *i* in the external archive and sub-population where individual *i* comes from, respectively. $N_{ib}^{SubPop}$ is the number of individuals that belongs to individual *i*’s sub-population and dominated by individual *i*. To minimize the value of $N_{ib}^{SubPop}$ can improve the quality of a sub-population. The less fitness value an individual has, the better the individual is, and also the better the sub-population to which the individual belongs is.
  8 $$Fit_{i} = 1 + N_{i}^{ExArchive} + N_{ia}^{SubPop} + N_{ib}^{SubPop}$$

The above fitness assignment strategy is combined with crowding distance^37^ to constrain the size of the external archive (*A*) within a predefined number ($\overline{N}$), and achieve a diversity and uniform Pareto front for our ATNFO problem.

Besides, an extending operator inspired by Ref.^33^ is executed. It reinserts a number of non-dominated individuals with large crowding distance into the evolving sub-population, and thus improves diversity and distribution of the obtained non-dominated solutions.

**Algorithm 2:**
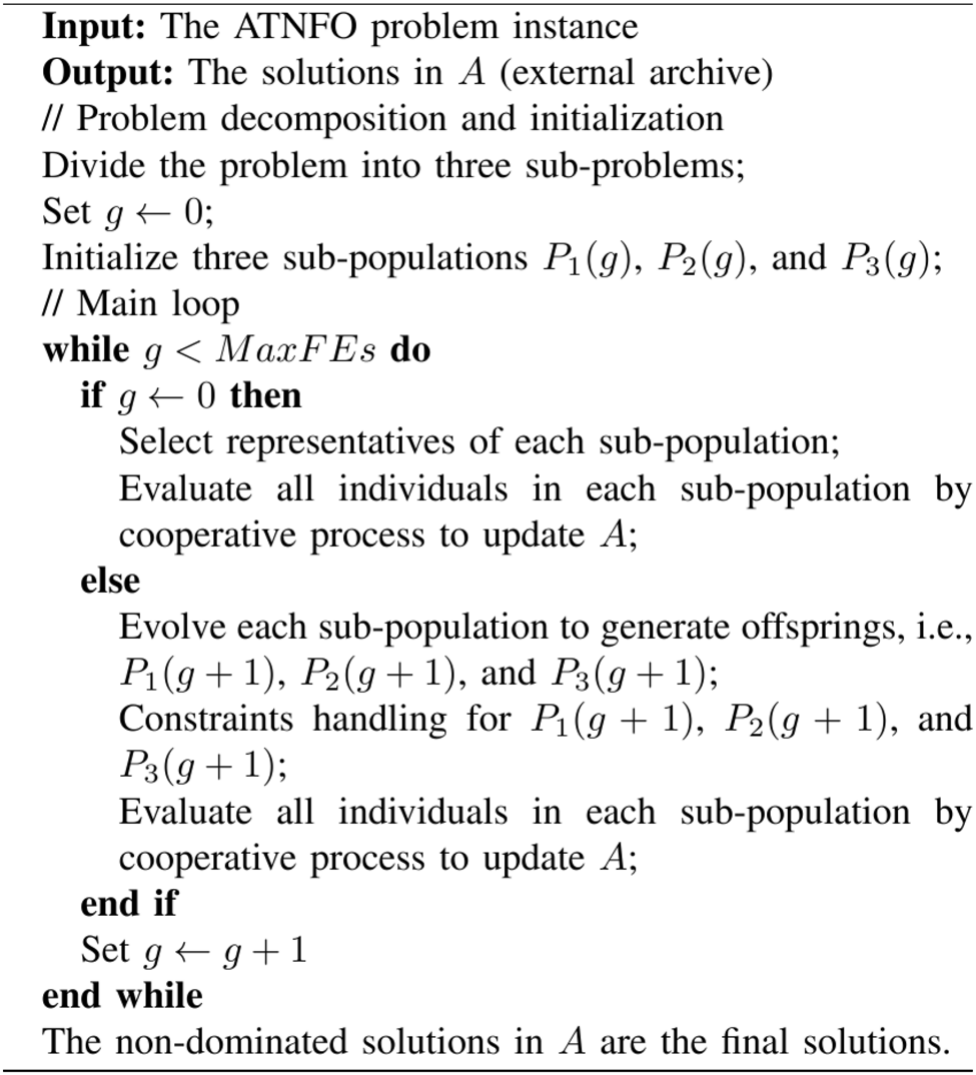
Pseudocode OF H-COEA.

### Full details of H-COEA

Let $MaxFEs$ be the maximum number of fitness evaluation in the whole optimization. Pseudocode of H-COEA is provided in Algorithm 2.

Details of evolving sub-populations and constraints handling in H-COEA for solving ATNFO problem are demonstrated as follows:

1. Sub-population evolving:
  (i) Selection: Select *N* individuals from current sub-population to form a “mating pool”, which used to generate offspring. The selection method shown as following: firstly, sort all individuals in current sub-population based on their fitness values and crowding distance values, then, select *N* individuals from current sub-population using binary tournament selection with crowed-comparison operator^38^. The crowed-comparison operator means that, for any two individuals, the one with lower fitness value will be selected. If both has the same fitness value, the one located in a lesser crowded region, i.e., has lager value of crowding distance, will be selected.
  (ii) Crossover and mutation operators: Apply crossover and mutation operators to “mating pool”, and get offspring sub-population*.* The chromosome in sub-population 3 has a sequence similar to a travelling salesman problem, whereby the timetabling row represents the order each flight is selected. Therefore, the choice of a crossover operator similar to those used for a travelling salesman problem, whereby feasibility of the children produced by crossover is guaranteed, is important. Hence, a Partially Matched Crossover (PMX) operator^39^ is applied to evolve sub-population 2 and 3 (i.e., the heuristic weight and timetabling), a uniform crossover operator^39^ is applied to evolve sub-population 1 (i.e., departure time-slot). Furthermore, a uniform mutation is applied to evolve sub-population 1 and 2 (i.e., the departure time-slot and heuristic weight) as in EqS. (9) and (10), respectively.
    9 $$D_{f} = \left\{ {\begin{array}{*{20}c} {D_{f} + U( - 2,2),ifrand_{f} < rate_{mutation} } \\ {D_{f} ,otherwise} \\ \end{array} } \right.$$
    10 $$H_{f} = \left\{ {\begin{array}{*{20}c} {H_{f} + U( - 0.1,0.1),ifrand_{f} < rate_{mutation} } \\ {H_{f} ,otherwise} \\ \end{array} } \right.$$
    where $U(a,b)$ is a uniform random value selected between *a* and *b*, $rand_{f}$ is a uniform random value selected between 0 and 1, and $rate_{mutation}$ is the mutation probability.
2. Constraints handling:
  During the sub-population evolving process, some individuals may violate constraints (EqS. (1)–(3)). Therefore, two constraint-handling techniques are applied here. Firstly, a repair procedure is designed to make sure all departure time-slots in new individual are feasible. If any departure time-slot violates the constraint (2), reset it within its search space randomly. This technique is only applied to evolve sub-population 1 (the departure time-slot). Secondly, if any flight route selected violates the constraint (3), the shortest path in ATN will be selected as its flight route. Thirdly, when an individual violates constraint (1), a penalty (Eq. (11)) will be enforced on objective 1 (Eq. (4)).
  11 $$\begin{gathered} f_{1} = \left\{ {\begin{array}{*{20}c} {f_{1} + penalty_{1} ,} & {if\,individual\,p\,violates\,constraint \,\left( 3 \right)} \\ {f_{1} ,} & {otherwise} \\ \end{array} } \right. \hfill \\ penalty_{1} = \sum\limits_{t = 1}^{T} {\sum\limits_{i = 1}^{n + m} {\left( {W_{t} \left( i \right) - C_{t} \left( i \right)} \right)^{2} } } \hfill \\ \end{gathered}$$
  $penalty_{1}$ means capacity violation will let the ATC workload increase sharply.

## Experimental study

In this section, experiments are carried out to analyze the performance of H-COEA for ATNFO problem, particularly its cooperative coevolutionary framework. By holding evolutionary operators constant and varying only the framework, we isolate the impact of cooperative coevolutionary paradigm on performance of solving ATNFO problems. A comparative study between H-COEA and NSGA-II, which are representatives of the state-of-the-art, is carried out to solve four different-scale ATNFO problems. Here, normal procedure in NSGA-II^38^ is employed, while chromosome representation and genetic operators are the same as H-COEA. All simulations are implemented in C +  + on a computer with a 3.20 GHz Intel(R) Core(TM) i5-3470 processor and a 4 GB RAM and under the Window 7 operating system.

The following subsections start with a description of ATNFO problems of various scales and performance metrics in Section "Different-scale instances generation" and Sect. “Performance metrics”, respectively. Subsequently, parameter settings will be analyzed in Sect. “Parameter settings”. And then, a comparative study including H-COEA and NSGA-II will be conducted in Sect. “Comparative study of H-COEA”.

### Different-scale instances generation

**Fig. 5:**
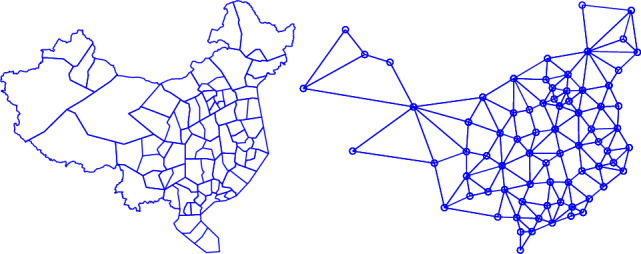
The sector configuration in the national airspace of China (left) and connection relations among sectors (right), circles represent sectors.

For all instances, the data of ATN are extracted from the national airspace of China in 2011. The airspace has been divided into 77 sectors and the connection relations among sectors are shown in Fig. 5. There are 150 airports, which supposed to connect with one sector where it located. The average crossing distance of sector is calculated according to the average length of air route that crosses the sector. The sectors and airports capacities are obtained from the historic data.

Except the data of ATN, the air traffic demand is the other necessary input data for solving an ATNFO problem. The air traffic demand is acquired from Flight Schedule Database (FSD) of summer and autumn in 2010 released by Civil Aviation Administration of China (CAAC). Each record in FSD indicates the scheduled flight information containing original airport, destination airport, scheduled departure/arrival time, etc.

Based on the real ATN data, we use various traffic demands to generate four different-scale ATNFO instances in the following experiments.

1. Instance 1, 526 flights scheduled during 8:00(am) to 9:00(am) on Friday to be assigned in the airspace.
2. Instance 2, 863 flights scheduled during 8:00(am) to 10:00(am) on Friday to be assigned in the airspace.
3. Instance 3, 1252 flights scheduled during 8:00(am) to 11:00(am) on Friday to be assigned in the airspace.
4. Instance 4, 1664 flights scheduled during 8:00(am) to 12:00(am) on Friday to be assigned in the airspace.

While this study uses a 2010 dataset for methodological validation, the proposed framework is designed to address modern challenges by leveraging scalable algorithms and invariant operational constraints. A validation on real-time 2023/2024 data (e.g., from China’s Civil Aviation Administration) is still significantly to assess performance under current traffic conditions. This will be explored in follow-up work.

### Performance metrics

To analyze the behavior of the compared algorithms for solving our bi-objective ATNFO problem, the Hypervolume metric (*I*_*H*_)^40^, the inverse generation distance (*I*_*D*_)^41^ and the spread ($\Delta$)^38^ of solution set are employed.

*I*_*H*_ measures the area of objective space dominated by obtained non-dominated solutions. The larger the *I*_*H*_, the closer the obtained set is to real Pareto-optimal front (POF, consists of Pareto-optimal solutions). *I*_*D*_ measures the average distance from the solution set to a reference set in objective space (Eq. (13)). If the reference set is defined as a set of Pareto-optimal solutions, a lower *I*_*D*_ value indicates a better convergence. $\Delta$ reflects the diversity of solutions in a population. Here we calculate $\Delta$ by the average Euclidian distance between all individuals in a non-dominated set. The details of calculating *I*_*H*_, *I*_*D*_ and $\Delta$ of a given non-dominated set *A* are shown as follows:

12 $$I_{H} (A) = \mathop {\int {...\int {} 1 \cdot dz} }\limits_{{z \in \mathop \cup \limits_{x \in A} HV(f(x),f^{*} )}}$$

13 $$I_{D} (A) = \frac{1}{\left| P \right|} \times \sum\limits_{i = 1}^{\left| P \right|} {(\min_{j = 1}^{\left| A \right|} d(i,j))}$$

14 $$\Delta (A) = \frac{{\sum\nolimits_{i = 1}^{\left| A \right| - 1} {d_{i} } }}{\left| A \right| - 1}$$

where $HV(f(x),f^{*} ) = [f_{1} (x),f_{1}^{*} ] \times ... \times [f_{q} (x),f_{q}^{*} ]$ is the Cartesian product of the closed intervals $[f_{q} (x),f_{q}^{*} ],i = 1,...,q$. $(f_{1}^{*} ,...,f_{q}^{*} )$ is a reference point and $f_{i}^{*} ,i = 1,...,q$ is usually defined as the worst value in non-dominated sets obtained by all compared algorithms for the minimization problem. For our ATNFO problem, we have $q = 2$, $f_{1} (x)$ and $f_{2} (x)$ are two objective values, i.e., ATC workload and total flight delays of solution *x*, respectively. In order to evaluate the performance of H-COEA during its whole evolution process, for each test instance in our experimental study, $f_{1}^{*} = \mathop {\max }\limits_{x \in \Omega } \{ f_{1} (x)\}$, $f_{2}^{*} = \mathop {\max }\limits_{x \in \Omega } \{ f_{2} (x)\}$, and $\Omega$ is defined as a set of all solutions obtained from all compared algorithms and over 30 runs. *P* is the set of Pareto-optimal solutions. |*P*| and |*A*| are numbers of solutions in *P* and *A* respectively. $d(i,j)$ is the Euclidean distance between the *i*^th^ solution in *P* to the *j*^th^ solution in *A*. $d_{i}$ is the Euclidean distance between the *i*^th^ left and the (*i* + 1)^th^ left solutions in *A*.

However, for ATNFO instance, it is difficult to obtain Pareto-optimal solutions. Alternatively, the non-dominated solutions obtained by both H-COEA and NSGA-II over 30 independent runs on a test instance are combined, and those solutions remained non-dominated in this set are used as the reference set for calculating *I*_*D*_. And moreover, in our experiments, the *I*_*H*_, *I*_*D*_, and ∆ are computed using normalized objective vectors.

### Parameter settings

Throughout our experiment, the average speed of flight (*v*) is set to 800; a time-slot is 2 min; the allowed adjustable range of departure time-slot is 15 time-slots; the allowed maximum flight time of flight is set as $t_{\max }^{f} = t_{\min }^{f} + 45,\,f\in F$ time-slots; the index $\varphi$ in objective *f*_1_ is set to 0.7; the weight *w* in objective *f*_2_ is set to 3.

About the algorithm, to find a better balance between exploration and exploitation, the crossover rate and mutation rate are analyzed in our H-COEA algorithm. Besides, the number (denoted as *ExtendNum*) of non-dominated individuals selected from the external archive when executing extending operator to improve diversity and distribution is also analyzed.

We test the effect of the above 3 parameters, i.e., crossover rate, mutation rate, and *ExtendNum*, by varying the crossover rate from 0.1 to 0.9, mutation rate from 0.001 to 0.009, and *ExtendNum* from 5 to 30 (the interval is 5). The three parameters are tested one by one. When analyzing one parameter, others are set as either expected value or default value. The default values of there 3 parameters are 0.8, 0.1, and 20, respectively. If we get one expected value, it will be used to test the remaining parameters. For all the test experiments, the *Instance 1* is used, the number of fitness evaluation is set to 10,000, and the sub-population size is set to 30.

**Fig. 6:**
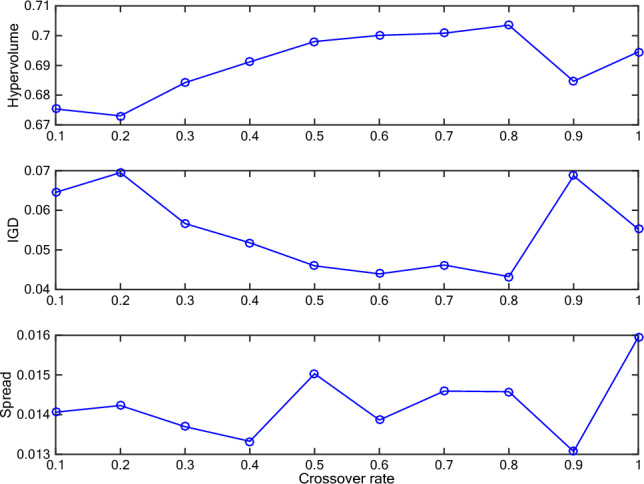
The obtained metrics with different crossover rate.

**Fig. 7:**
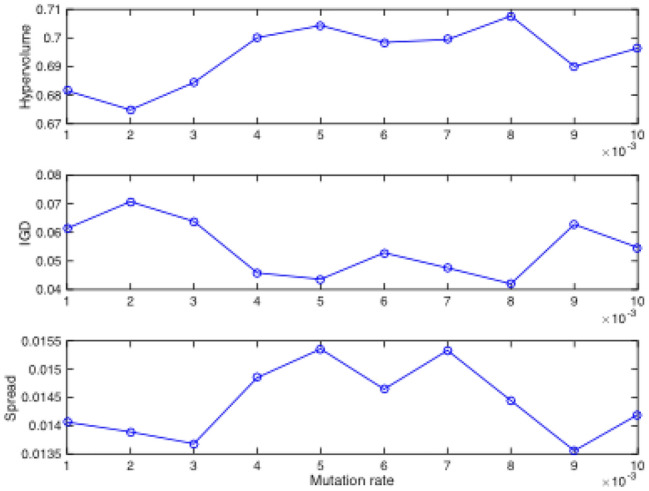
The obtained metrics with different mutation rate.

**Fig. 8:**
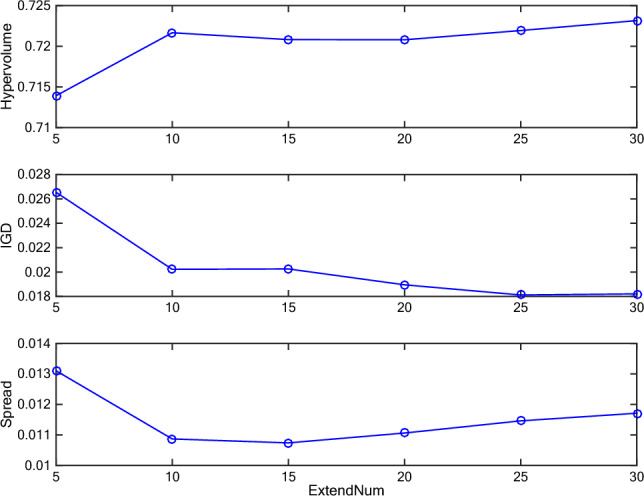
The obtained metrics with different *ExtendNum.*

During the test experiment, metrics, i.e., *I*_*H*_, *I*_*D*_, and ∆, are recorded to assess the performance. To calculate these metrics, all obtained non-dominated sets were combined, and those solutions remained non-dominated in the combined set were used as the reference set, i.e., the Pareto optimal front. The largest values of two objectives are selected from all solutions, which obtained from all algorithms and over 30 independent runs, for calculating *I*_*H*_. Figures 6, 7 and 8 depict the obtained metrics with different crossover rate, mutation rate, and *ExtendNum*. It can be noted that the best performance is obtained with the crossover rate of 0.8, the mutation rate of 0.008, and the *ExtendNum* of 10.

Based on the above analysis, the parameter settings of H-COEA are set as Table 1 in the following comparative study. To be fair, the parameter settings of the comparative algorithm, i.e., the NSGA-II with chromosome representation and genetic operators the same as H-COEA, are set as Table 1 according to our previous work in Ref.^8^, which they were rigorously validated in similar experimental settings.

**Table 1:** Parameter settings of H-COEA and NSGA-II.

Parameters	H-COEA	NSGA-II
Population size	30	100
Number of sub-populations	3	N/A
Number of fitness evaluation	10,000	10,000
Crossover rate	0.8	0.8
Mutation rate	0.008	0.009
*ExtendNum*	10	N/A
External archive size	100	N/A

Unless mentioned otherwise, all results are collected based on 30 independent runs to eliminate the stochastic behavior that may be caused by the random generator.

### Comparative study of H-COEA

Firstly, the average value of *I*_*H*_, *I*_*D*_, and ∆ with H-COEA and NAGA-II for solving four different-scale ATNFO problems over the 30 independent runs are presented in Table 2. All corresponding standard deviations are given in parenthesis. The best value in each row is highlighted in boldface. For each metric, the Wilcoxon rank sum test^42^ has been carried out to compare H-COEA with NSGA-II. If an algorithm performs significantly better than the other one with respect to a performance metric, the corresponding result will be highlighted with “*”. The stared *I*_*D*_ and ∆ are the smaller while *I*_*H*_ is the greater one between H-COEA and NSGA-II. Table 2 implies, in terms of *I*_*H*_, *I*_*D*_, and ∆, H-COEA achieves the better performance and is significantly superior to NSGA-II when solving larger scale ATNFO problem (such as *Instance 2*, *3*, and *4*).

**Table 2:** The obtained metrics with H-COEA and NSGA-II for solving four different-scale ATNFO problems.

Instances		*H-COEA*	*NSGA-II*
*Instance 1*	*I*_*H*_	0.5884 (0.0634)	**0.6893** (0.0090)*
	*I*_*D*_	0.1063 (0.0589)	**0.0281** (0.0066)*
	$\Delta$	0.0247 (0.0087)	**0.0217** (0.0084)*
*Instance 2*	*I*_*H*_	**0.5406** (0.0149)*	0.4901 (0.0050)
	*I*_*D*_	**0.0346** (0.0220)*	0.0921 (0.0027)
	$\Delta$	**0.0090** (0.0019)*	0.0104 (0.0017)
*Instance 3*	*I*_*H*_	**0.3939** (0.0620)*	0.3510 (0.0104)
	*I*_*D*_	**0.0985** (0.0947)*	0.1206 (0.0035)
	$\Delta$	**0.0088** (0.0040)*	0.0129 (0.0025)
*Instance 4*	*I*_*H*_	**0.4737** (0.0559)*	0.3494 (0.0045)
	*I*_*D*_	**0.0748** (0.0713)*	0.1780 (0.0038)
	$\Delta$	**0.0077** (0.0036)*	0.0106 (0.0017)

The best value in each row is in bold.

**Fig. 9:**
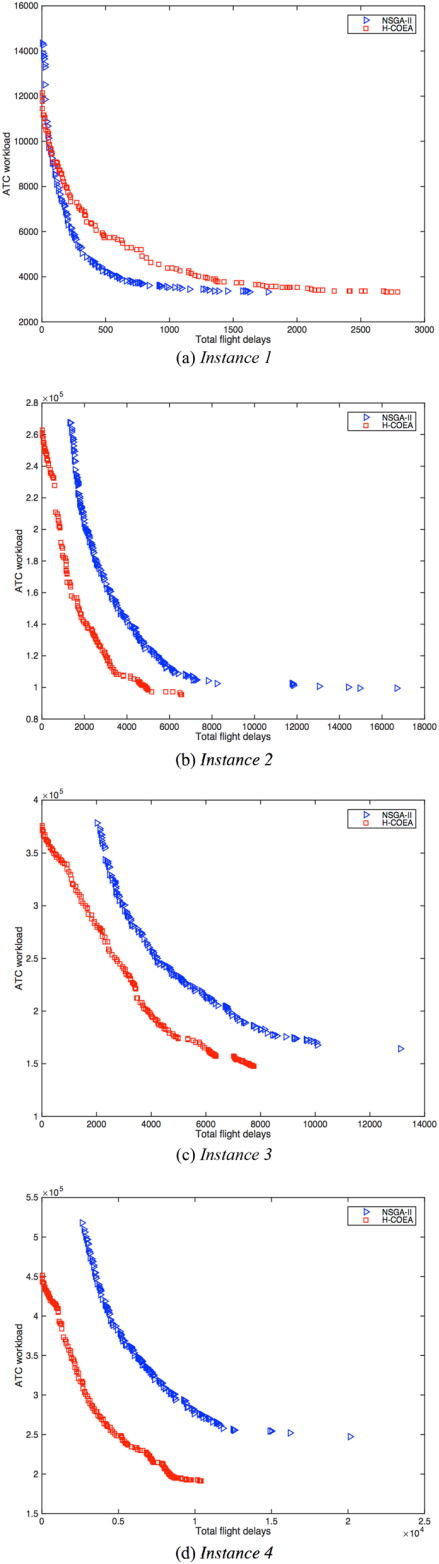
The obtained Pareto fronts with H-COEA and NAGA-II for solving four different-scale ATNFO problems. (**a**) Comparisons between H-COEA and NAGA-II for solving the *Instance 1.* (**b**) Comparisons between H-COEA and NAGA-II for solving the *Instance 2.* (**c**) Comparisons between H-COEA and NAGA-II for solving the *Instance 3.* (**d**) Comparisons between H-COEA and NAGA-II for solving the *Instance 4.*

Furthermore, the obtained Pareto fronts with H-COEA and NAGA-II for solving four different-scale ATNFO problems are plotted in Fig. 9. It is obviously that our H-COEA performs better than NSGA-II (except for solving *Instance 1*, see Fig. 9a). And, as the scale of ATNFO problem increases, the distance between the Pareto front obtained by H-COEA and that obtained by NAGA-II becomes larger and larger. According to the decision-makers’ preferences, one can get a better choice from the Pareto front obtained by H-COEA. It means the CC paradigm is more suitable for solving large scale ATNFO problem. In practical air traffic flow management, the scale of ATNFO problem is usually large, for example, 20,000 flights fly over the airspace of China in a day and are needed to consider now.

In summary, the proposed approach rigorously establishes the effectiveness of the coevolutionary framework, positioning H-COEA as a novel contribution beyond incremental improvements to existing algorithms. Moreover, H-COEA’s framework can be integrated with any evolutionary operators, offering a versatile improvement over existing algorithm.

## Conclusion

The practical important bi-objective nonlinear ATNFO problem is invested in this paper, and an approach based on CC paradigm, i.e., H-COEA, is employed to explore the optimized trade-offs between safety and efficiency for it. Unlike previous work, H-COEA is a combination of CC paradigm and an effective chromosome representative scheme, which can be naturally divided into 3 sub-components, i.e., the departure time-slots, the heuristic for selecting flight route, and the timetabling indicating the order for flight to select route. Comprehensive experiments have been carried out using real data of the Chinese airspace and four different-scale sets of flights to be assigned in airspace. The experimental results demonstrate the advantage of H-COEA over the best-known NSGA-II as the scale of ATNFO problem increases. The utility of H-COEA is further justified by the fact that it managed to both increase the airspace safety of the national ARN of China (i.e., reduce the ATC workload) and reduce the total flight delays simultaneously.

Since the mathematic model does not explicitly account for hierarchical interactions between safety and effectiveness objectives (e.g., bi-level dynamics in ATC workload and airspace capacity). Future work could explore bi-level programming or game-theoretic frameworks to better capture such dependencies, though this would introduce significant computational challenges.

## Data Availability

The data presented in this study are available through email upon request to the corresponding author.
